# Effect of pequi (*Caryocar brasiliense*) and juçara (*Euterpe edulis*) waste extract on oxidation process stability in broiler meat treated by UV-C

**DOI:** 10.1371/journal.pone.0208306

**Published:** 2018-12-20

**Authors:** Beatriz Frasao, Marion Costa, Fabricio Silva, Bruna Rodrigues, Jéssica Baltar, Jasmim Araujo, Daniel Moreira, Renata Torrezan, Carlos Conte-Junior

**Affiliations:** 1 Collegiate of Veterinary Medicine, Multidisciplinary Center of Barra *Campus*, Universidade Federal do Oeste da Bahia, Barra, Bahia; 2 Department of Preventive Veterinary Medicine and Animal Production, Escola de Medicina Veterinária e Zootecnia, Universidade Federal da Bahia, Ondina, Salvador; 3 Food Science Programe, Chemistry Institute, Universidade Federal do Rio de Janeiro, Rio de Janeiro, Brazil; 4 Department of Food Technology, Faculdade de Veterinária, Universidade Federal Fluminense, Rio de Janeiro, Brazil; 5 Embrapa Food Technology, Brazilian Agricultural Research Corporation, Rio de Janeiro, Brazil; 6 National Institute for Health Quality Control, Oswaldo Cruz Foundation (FIOCRUZ), Rio de Janeiro, Brazil; Tallinn University of Technology, ESTONIA

## Abstract

The aim of this study was to determine the potential for waste extracts from the pequi (*Caryocar brasiliense*) and juçara (*Euterpe edulis*) to reduce oxidatiove processes in antibiotic-free broiler meat. The use of natural antioxidants extracted from fruit-processing wastes has been neglected. Although these residues contain high amounts of these bioactive compounds, they are often discarded by industry. Meat samples were exposed previously submitted to UV-C radiation at 1.161 mW / cm^2^ for 10 minutes to accelerate the rancidity process. Pequi and juçara waste extracts were obtained by microwave-assisted extraction (MAE). A total of four conditions were tested using antibiotic-free broiler thighs and drumstick meat: BN–with no antioxidant (negative control), BP–with BHT (Butylated hydroxytoluene) (positive control), BE–with juçara extract, BC–with pequi extract. The color, pH, lipid and protein oxidation (days 0, 2, 4, 6, 8 and 10), antioxidant contents and activity (days 0 and 10), and proximal composition and fatty acid profile (day 0) were tested, followed by principal component analysis (PCA). Pequi waste extract presented the highest antioxidant content and activity. BE and BC treatments presented the highest total phenolic (TPC) and flavonoid (TFC) content, and BE presented the highest total monomeric anthocyanin content (TAC). TFC increased during storage in all treatments. The waste extracts of *C*. *brasiliense* presented the highest antioxidant activity against lipid oxidation in the antibiotic-free broiler meat. Moreover, both extracts presented high antioxidant activity against protein oxidation. Although the pequi peel extract had a better effect in terms of suppressing both types of oxidation, either this extract or the jussara waste extract could be used as a technological strategy to reduce the oxidative processes in antibiotic-free broiler meat for the poultry industry. Thus, waste extracts can be a potential technology to reduce the oxidative processes in antibiotic-free broiler meat.

## Introduction

Oxidation is a major cause of chicken meat spoilage during storage [1]. Thus, lipid and protein oxidation are directly related to deterioration and reduced shelf life in minced meat products. Lipid oxidation is initiated in the unsaturated fatty acids fraction and forms hydroperoxides, which are susceptible to oxidation [2]. This process produces changes in the meat quality parameters and also decreases the nutritional value. In protein oxidation, covalent modification of protein is induced either directly by reactive species or indirectly by reaction with secondary by-products of oxidative stress [3]. However, oxidation in meat products can be controlled or minimized using food additives, such as antioxidants [4].

The food industry usually uses synthetic antioxidants such as butylated hydroxyanisole (BHA), butylated hydroxytoluene (BHT), and tertiary butyl hydroquinone (TBHQ) to reduce or minimize the oxidative process in meat [5]. However, these chemicals can present toxicological and carcinogenic effects [5,6]. Therefore, consumers have increasingly come to prefer natural preservatives [6]. In addition, the use of natural antioxidants has better consumer acceptance [7] due to health benefits. However, few studies have reported on the use of natural antioxidant-rich fruit extracts as inhibitors of lipid and protein oxidation to extend the shelf life and improve the quality of chicken meat products [6,8–10].

The juçara (*Euterpe edulis*) and pequi (*Caryocar brasiliense*) are native Brazilian fruit originating in Mata Atlântica and Cerrado, respectively [11,12]. Both present high concentration of antioxidant compounds, such as phenolic acids, flavonoids and carotenoids, presenting high antioxidant potential. [13,14]. The extract of the juçara showed antioxidant activity that was correlated to the presence of high concentration of phenolic acids, flavonoids and anthocyanins, the last one is the main pigment responsible for its purple coloration [14–17]. However, the extract of pequi presents antioxidant activity that was correlated to the presence of phenolic compounds, flavonoids and carotenoids [18–20]. These fruits are usually marketed in the form of pulp, in which they are discarded. For example, the outer peel and mesocarp of pequi account for 70% of the fruit [21].

For these reasons, the aim of this study was to evaluate the antioxidant capacity of waste extracts from the processing of Brazilian native fruits (*Caryocar brasiliense* and *Euterpe edulis*) for the inhibition of the oxidation in antibiotic-free broiler meat and examine any possible color changes. As a pretreatment stage, UV-C radiation was applied to stimulate oxidation to further test the antioxidant effect.

## Materials and methods

### Chemical compounds

Thiobarbituric acid (PubChem CID: 2723628); 2,4-dinitrophenylhydrazine (DNPH-PubChem CID: 3772977); gallic acid (PubChem CID: 46780424); quercetin (PubChem CID: 5280343); cyanidin 3-O-glucoside (PubChem CID: 12303203); beta-carotene (PubChem CID: 5280489); linoleic acid (PubChem CID: 5280450); guanidine (PubChem CID: 3520); and malonaldehyde (PubChem CID: 10964).

### Determination of UV-C radiation time

To determine the time of exposure to UV-C radiation that was necessary to induce oxidation in the chicken meat, four treatments were performed: UV-C exposure at 1.161 mW/cm^2^ for 5 minutes (5I), 10 minutes (10I), 15 minutes (15I) or control without irradiation (WI). In addition, lipid oxidation in broiler meat (with and without exposure) was evaluated for 10 days at 4±1°C. For the application of UV-C light, a previously constructed stainless-steel barrel-shaped chamber [22] was used.

### Sources of pequi and juçara waste

Pequi (*Caryocar brasiliense*) fruit was collected from Montes Claros-MG (16° 44' 06" S, 43° 51' 42" W), in January and February 2016. The epicarp and external mesocarp of pequi were separated. The epicarp and endocarp of juçara (*Euterpe edulis*) berries were supplied by Juçaí Industry (Juçaí, Rio de Janeiro, Brazil, 22° 240 4400 S, 42° 570 5600 W) in May 2015. The samples were blanched and immediately frozen at -20°C to inactivate enzymes while maintaining the fruit’s general properties. The wastes were air-dried at 60°C in the oven with forced air circulation (330 drier, FANEM, Brazil) until they reached a constant weight (9 h) and then ground in a mill (A11 Bsic, IKA Werke, Staufen, Germany). The ground samples were sieved through a 250 Mesh screen because particle size affects the extractability of bioactive molecules [23]. All samples were stored at -20°C until further use.

### Pequi and juçara waste extract preparation

The extracts were obtained by microwave-assisted extraction (MAE) utilizing a DGT 100 Plus system (Provecto Analytics Ltd., Jundiaí, SP, Brazil). Briefly, 500 mg aliquots of the fruit powder were added to 25 mL of an aqueous ethanol solution (94%, v/v), sealed in extraction vessels, and subjected to extraction at 670 W microwave power for 110 seconds. After each extraction, the vessels were cooled to 25°C before centrifugation at 1,400 × *g* for 10 min at 4°C. Each precipitate was re-extracted with an additional 25 mL of the same ethanol solution under the same MAE conditions; the supernatants were pooled and stored in amber vials at -20°C. Analyses of the total phenolics (TPC), flavonoids (TFC) and monomeric anthocyanins (TAC), as well as antioxidant activity were analyzed for the two extracts. TPC was used to dose the volume of extracts applied in the broiler meat treatments (100 mg/kg).

### Broiler meat samples preparation

Five kilograms of antibiotic-free (AF) broiler thighs and drumsticks were obtained from Korin natural agriculture. The broiler thighs and drumsticks were transported under refrigerated conditions. The samples were boned, and meat with skin was ground (8 and 6 mm). For standardization, 20% (w/w) of broiler fat was added. A thin layer (0.5 cm) of mixed meat and fat were exposed to UV-C radiation (1.195 mW/cm^2^ for 10 minutes) to induce oxidation in the meat.

Subsequently, 100 mg/kg of each extract or BHT was added for the various treatments. For the negative control, no antioxidant was added (BN–broiler negative control); for the positive control a synthetic antioxidant was added (BP–broiler positive control (BHT)); for the treatments with fruit, *Euterpe edulis* (BE–broiler *Euterpe*) and *Caryocar brasilense* (BC–broiler *Caryocar*), the waste extracts were added. All samples were mixed for one minute with a food mixer, samples of 30 g each were prepared. All samples were packaged under aerobic conditions in polyethylene bags and sealed with a vacuum-packaging machine (TECMAQ, São Paulo, Brasil) Vacuum sealer, AP 450). The experiment was performed in triplicate.

The samples were stored at 4±1°C for 10 days. Color, pH, lipid and protein oxidation analyses were performed on days 0, 2, 4, 6, 8 and 10. The antioxidant analysis was carried out on days 0 and 10 for the samples and for the extracts. Proximal composition and fatty acid profiles were tested on day 0, for characterization. All analyses were performed in triplicate.

### Analytical methods for antioxidants

Total phenolic content (TPC), total flavonoid content (TFC) and total monomeric anthocyanin content (TAC) of the *E*. *edulis* waste extracts and broiler treatments were estimated based on the Folin–Ciocalteu method at 765 nm [24], a colorimetric method at 415 and 700 nm [25], and the pH differential (pH 1.0 and pH 4.5) method at 520 and 700 nm [26], respectively. The absorbance values were measured using a UV-1800 spectrophotometer (Shimadzu Corporation, Kyoto, Japan). The results were expressed as mg gallic acid equivalents (GAE) per mL of extract, mg of quercetin equivalents (QE) per mL of extract, and mg cyanidin-3-glucoside equivalents per liter of extract.

A β-carotene bleaching assay was used to determine the antioxidant activity of the extracts with a modified β-carotene-linoleic acid model system modified by [15], using a UV-1800 Spectrophotometer (Shimadzu Corporation, Kyoto, Japan). The antioxidant activity of extracts was expressed as a percentage.

### Proximate composition

Moisture (reference 940.05) was determined by the stove method at 105°C [27]. The protein content (reference 954.01) was determined by the micro-Kjeldahl method [27]. The ash content (reference 942.05) was determined by total carbonization of the sample [27]. The lipid content was determined as previously described [28]. The pH of the samples was measured using a digital potentiometer (model PG1800, Cap Lab, SP, Brazil).

### Fatty acid profile

To determine the fatty acid profile, a cold extraction of total lipids from the samples was performed in quadruplicate based on a previously described method [28] with slight modifications, as previously described. Methylation was achieved through an acidic reaction (10% HCl in methanol) with hexane [29,30].

The fatty acid methyl esters (FAME) were analyzed using a gas chromatograph equipped with a flame ionization detector (Perkin Elmer, Waltham, MA, USA) and separated with an OmegawaxTM 320 column (30 m length, 0.32 mm internal diameter, and 0.25 μm particle size) (Supelco Inc., Bellefonte, PA, USA). The sample size was 2 μL, and the split used was 1:20. The injector and detector temperatures were set at 260°C and 280°C, respectively. The initial temperature of the oven was set at 110°C, and the temperature ramp was as follows: increased from 110 to 233°C at 40°C/min, held at 233°C for 2 min, increased from 233 to 240°C at 1°C/min, and held at 240°C for 21 min. Helium was used as the carrier gas at a flow rate of 1.8 mL/min (10 psi). The FAME were identified by comparisons with the retention times for a commercial standard of the methyl esters of 28 individual fatty acids (Supelco Inc., Bellefonte, PA, USA). The results were expressed in percentages, as proposed by Memon et al. [31].

### Lipid oxidation

To determine the lipid oxidation, the secondary products of lipid oxidation were measured using the UV-1800 spectrophotometer (Shimadzu Corporation, Kyoto, Japan) at 532 nm by quantification of substances reactive to thiobarbituric acid (TBARS) at the various time points during storage using the methodology modified by Alcântara et al. [32]. The results were expressed as mg of malonaldehyde (MDA) per gram of meat, based on a calibration curve.

### Protein oxidation

To determine the protein oxidation, the total carbonyl content was estimated utilizing a 2,4-dinitrophenylhydrazine (DNPH) derivatization assay based on a previously modified method [33]. The protein content was estimated on the basis of the absorbance at 280 nm compared with a bovine serum albumin standard curve, whereas the carbonyl content was calculated from the absorption at 370 nm using an absorptivity coefficient for protein hydrazones of 21.0 mM^-1^ cm^-1^. The results were expressed as nmol of carbonyl per mg of protein.

### Color evaluation

Color determinations were made at 10°C using a Minolta CM-600D spectrophotometer (Minolta Camera Co., Osaka, Japan). Natural broiler samples were exposed to oxygen for 5 minutes, and the sensor was mounted directly on the upper surface of the patties to prevent ambient light noise [34]. The following color CIELab coordinates were determined: lightness (L*, 100 = white, 0 = black), redness (a*, + red, −green), and yellowness (b*, + yellow, −blue). In addition, the total color change ΔE*, hue angle h°, and chroma C* were also calculated [35].

### Statistical analysis

The results are represented as the means ± standard deviation. Analyses of variance (ANOVA) and Tukey’s test were performed to evaluated the differences. Differences were considered to be significant at p < 0.05. Principal component analysis (PCA) was applied to visualize the parameters affected by the addition of the antioxidants on days 0 and 10 of storage at 4°C. Pearson correlation analysis was performed to examine the data (p < 0.05). All statistical analyses were performed using XLSTAT version 2013.2.03 software (Addinsoft, Paris, France).

## Results and discussion

### Determination of UV-C radiation time

**Fig 1:**
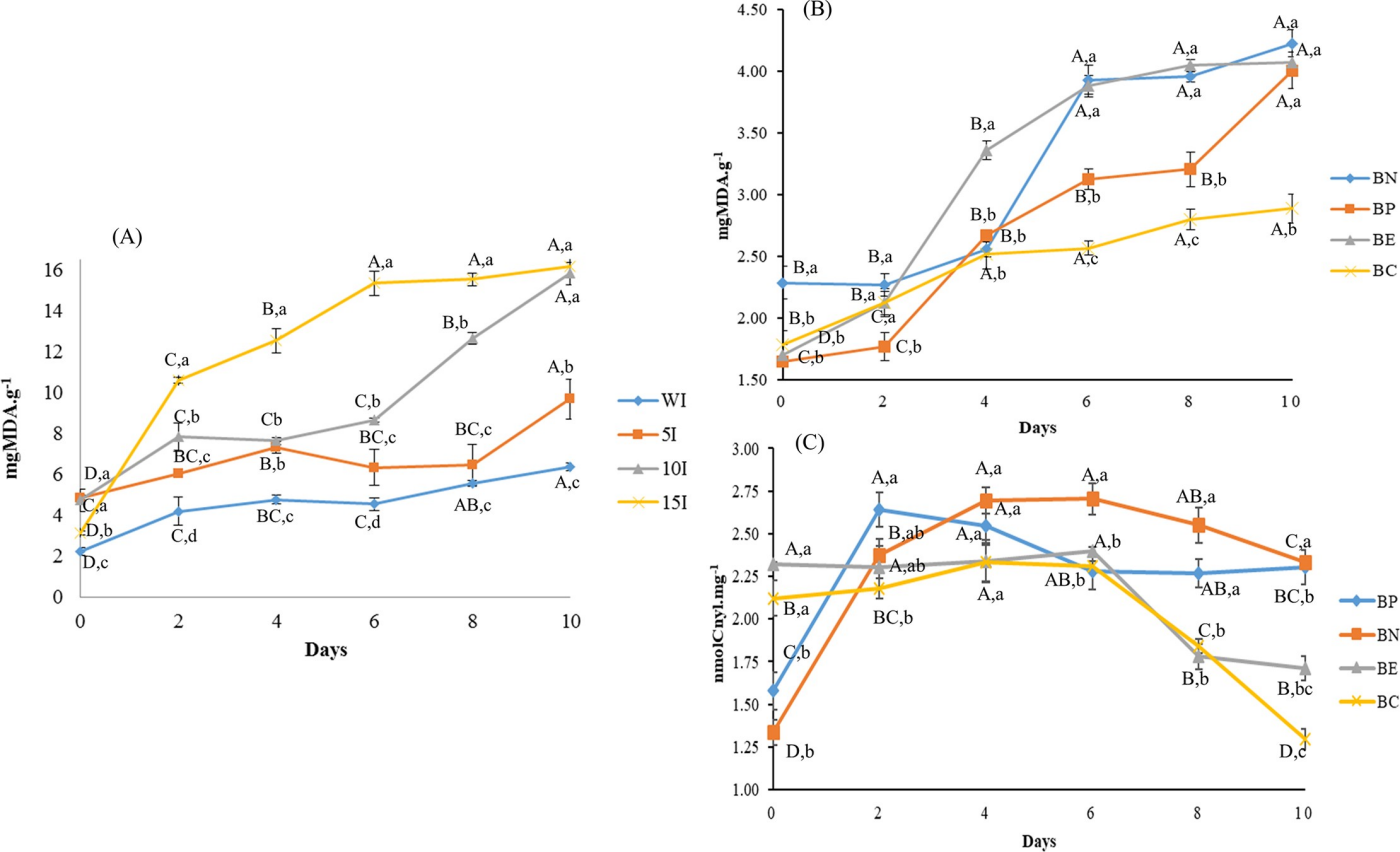
**Lipid oxidation of broiler meat subjected to UV-c irradiation for various times (A), and oxidation of broiler meat treatments during storage at 4**°**C, for 10 days. (B) Lipid oxidation. (C) Protein oxidation.** All results are shown as the means with standard deviation (n = 3). a-d: the same letters indicate no difference between treatments on the same day results; different letters indicate that were differences. A-D: the same letters indicate no difference between the days; different letters indicate that were differences. WI–broiler meat without irradiation; 5I –broiler meat with 5 minutes irradiation; 10I –broiler meat with 10 minutes irradiation; 15I –broiler meat with 15 minutes irradiation.

Unexposed meat (WI) showed lower initial values of lipid oxidation (p < 0.05) than the others (5I, 10I and 15I) (Fig 1A), which confirms that the pre-exposure of chicken meat to oxidation due to UV-C radiation caused oxidation. However, only the I10 and I15 treatments presented greater lipid oxidation (P < 0.05) than the control from the beginning to the end of the storage period. Therefore, the I10 treatment was sufficient to stimulate oxidation in chicken meat.

### Antioxidant compounds and activity from natural extracts

The TPC, TFC and TAC contents of extracts from the wastes of *Caryocar brasiliense* and *Euterpe edulis* contents are shown in Table 1. The *C*. *brasiliense* extract presented the highest levels of TPC, TFC and TAC than the *E*. *edulis* extract (Table 1), which indicated that the pequi peel has higher concentrations of these compounds than the juçara waste (p<0.05). However, both extracts presented satisfactory levels of natural antioxidant compounds compared to other studies that used extraction conditions similar to our study [15,36].

**Table 1:** Antioxidant content and antioxidant activity of pequi (Caryocar brasiliense) and juçara (Euterpe edulis) waste extracts.

Extracts	TPC^a^	TFC^b^	TAC^c^	Antioxidant Activity ^d^				
				0 min	30 min	60 min	90 min	120 min
***Caryocar brasiliense***	3.77±0.14 ^A^^,^^a^	1.64±0.01 ^B^^,^^a^	0.92±0.08 ^C^^,^^a^	100.00±0.10 ^A^^,^^a^	100.00±0.12 ^A^^,^^a^	96.96 ±0.05 ^B^^,^^a^	93.01 ±0.13 ^C^^,^^a^	90.44 ±0.11 ^C^^,^^a^
***Euterpe edulis***	0.34±0.00 ^A^^,^^b^	0.24±0.00 ^B^^,^^b^	0.21±0.04 ^C^^,^^b^	100.00±0.05 ^A^^,^^a^	90.48±0.10 ^B^^,^^b^	85.11 ±0.11 ^B^^C^^,^^b^	81.74 ±0.12 ^C^^D^^,^^c^	78.42 ±0.12 ^D^^,^^c^
**BHT**	-	-	-	100.00±0.02 ^A^^,^^a^	92.04 ±0.02^B^^,^^b^	89.82±0.10 ^C^^,^^b^	86.45±0.12 ^D^^,^^b^	83.74±0.11^E^^,^^b^

All results are means ± SD (n = 3).

^a^(mgGAE.mL^-1^)

^b^(mgQE. mL ^-1^)

^c^(mgC3QE. L ^-1^)

^d^(AA%)

a-c Same letters indicate no difference between lines results; different letters indicate difference.

A-E Same letters indicate no difference between columns results; different letters indicate difference.

BHT is a positive control used to determine antioxidant activity.

Regarding antioxidant activity, at initial time (0), both extracts presented the same activity (100%) (p>0.05). However, as early as 30 minutes until the end of the analysis (120 min), the *C*. *brasiliense* waste extract had a higher (p<0.05) antioxidant activity (Table 1). This result was associated with the grater concentrations of antioxidant compounds in this extract compared to the *E*. *edulis* waste extract, despite the use of the identical extraction parameters. This positive relationship between the concentration of phenolic compounds and antioxidant activity has been observed by other authors [15,36,37].

### Broiler meat characterization

On proximate composition analysis of the composition (Table 2), no differences were observed among the treatments (BN, BP, BE and BC) (Table 2). The addition of extracts did not influence these parameters because they consist primarily of antioxidant compounds. Therefore, in this study, the proximate composition of the treatments (BN, BP, BE and BC) cannot be considered a biasing factor.

**Table 2:** Proximate composition of broiler meat treatments.

Treatments	Moisture	Protein	Ash	Lipid
**BN**	67.35±0.75^a^	19.45±0.17^a^	0.96±0.01^a^	8.01±0.08 ^a^
**BP**	67.79±0.14^a^	19.44±0.53^a^	0.96±0.02^a^	8.16±0.06 ^a^
**BE**	69.20±0.51^a^	19.85±0.01^a^	0.94±0.01^a^	7.95±0.08 ^a^
**BC**	69.00±0.14^a^	20.37±0.51^a^	0.98±0.01^a^	8.00±0.04 ^a^

All results are means ± SD (n = 3).

Means that do not share a letter are significantly different.

BN–broiler negative control; BP—broiler positive control (with BHT); BE–broiler meat with *E*. *edulis*; BC–broiler meat with *C*. *brasilience*

**Fig 2:**
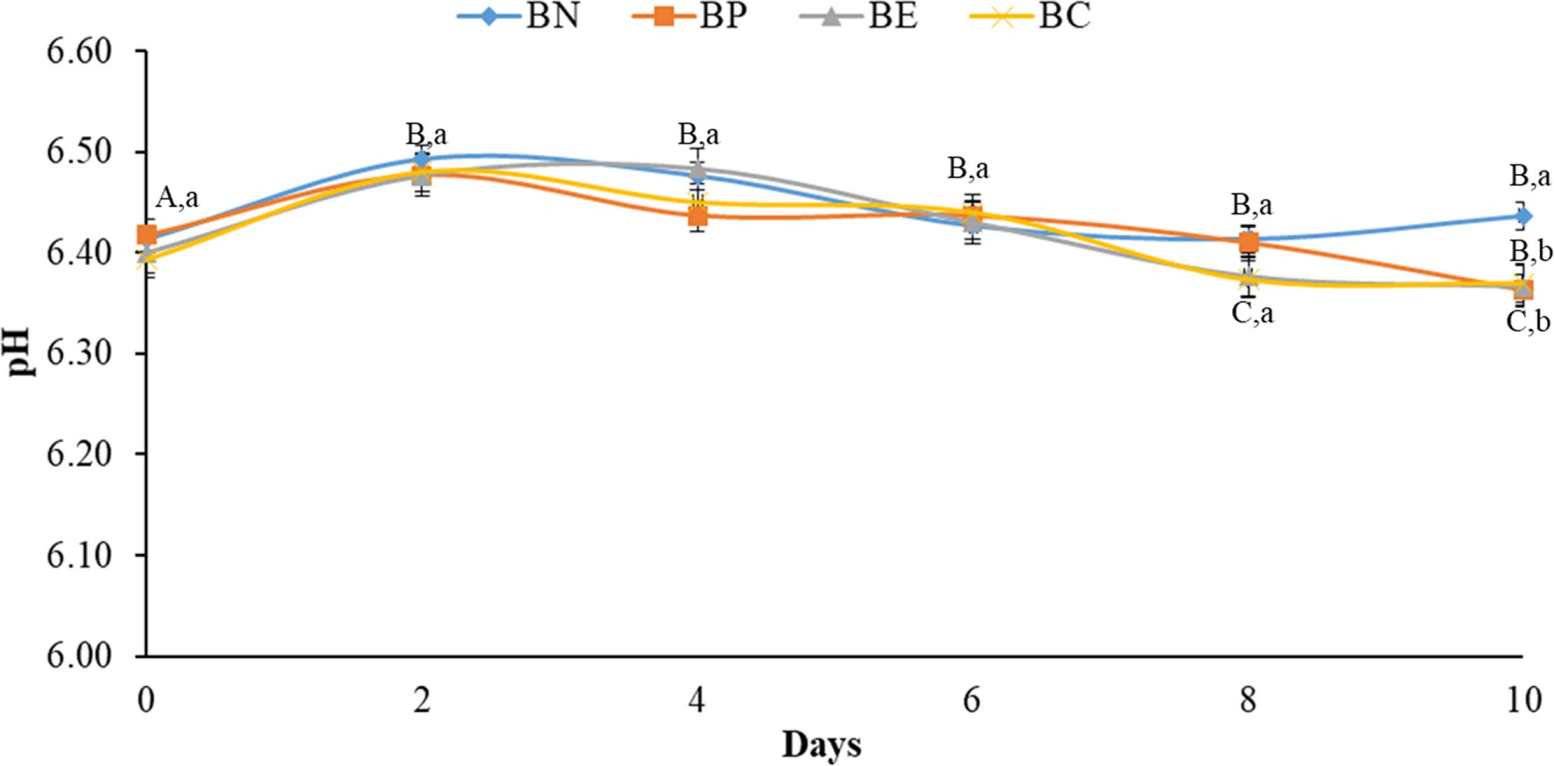
pH values of treated broiler meat during storage at 4°C, for 10 days. All results are means with standard deviation (n = 3). a-b: the same letters indicate no difference between treatments in same day results within the analysis; different letters indicate a difference. A-C: the same letters indicate no difference in results between the days within the analysis; different letters indicate a difference. BN–broiler negative control; BP—broiler positive control (with BHT); BE–broiler meat with *E*. *edulis*; BC–broiler meat with *C*. *brasiliense*.

The BC showed a higher pH value, whereas the samples treated with synthetic and natural antioxidants (BP, BE and BC) (Fig 2) showed no differences (p>0.05) between the initial and final pH values. However, the pH only differed (p<0.05) between the treatments (BP, BE and BC) and BC on the last day of storage; BC presented the highest pH value. This finding can be explained by the fact that antioxidants also act as general preservatives [10,38] that inhibiting the deterioration process.

The fatty acid profile of the raw material (chicken meat) is presented, in g/100 g is shown in Table 3. A similar characteristic in this profile was also observed previously by Dalziel, Kliem and Givens [39]. The content of mono and polyunsaturated fatty acids in a meat matrix interferes with lipid oxidation potential [2].

**Table 3:** Fatty acid profile of the broiler meat matrixes.

Fatty acids	Antibiotic-Free Broiler Meat
**C6:0**	2.52±0.29
**C8:0**	0.27±0.02
**C10:0**	0.67±0.07
**C11:0**	0.31±0.01
**14:0**	48.69±4.52
**16:0**	11.67±1.42
**16:1**	1.09±0.38
**17:0**	1.47±0.09
**18:0**	0.54±0.10
**18:1n7**	6.39±0.96
**18:1n9**	8.29±0.89
**18:2n6**	9.87±1.40
**18:3n6**	0.92±0.12
**18:3n3**	0.17±0.02
**20:1**	0.16±0.01
**20:2**	0.13±0.01
**20:3n6**	2.91±0.48
**20:4n6**	2.29±0.50
**22:2**	0.36±0.01
**DHA (22:6n3)**	0.14±0.01
**∑ SFA**	67.28±0.21
**∑ MUFA**	15.94±0.11
**∑ PUFA**	16.78±0.10

SFA = saturated fatty acid; MUFA = monounsaturated fatty acid; PUFA = polyunsaturated fatty acid; DHA = docosahexaenoic acid

All results are means ± SD (n = 3), g/100 g fatty acids.

Means that do not share a letter are significantly different.

### Antioxidant compounds in broiler meat treatments

The results of TPC, TFC and TAC in broiler meat treatments (BP, BE and BC) during refrigerated storage time (0 and 10 days) are shown on Table 4.

**Table 4:** Antioxidant contents in treated broiler meat during storage at 4°C for 10 days.

Treatments	TPC (mg AGE.100 g^-1^)		TFC (mg QE.100 g^-1^)		TAC (mg C3GE.100 g^-1^)	
	**Day 0**	**Day 10**	**Day 0**	**Day 10**	**Day 0**	**Day 10**
**BN**	58.57±3.05 ^A^^,^^b^	45.46±4.20 ^B^^,^^b^	81.11±1.62 ^B^^,^^c^	333.07±3.66 ^A^^,^^b^	3.06±0.04 ^A^^,^^b^	0.96±0.05 ^B^^,^^c^
**BP**	70.81±5.36 ^A^^,^^a^^b^	49.52±4.96 ^B^^,^^b^	205.39±4.05 ^B^^,^^b^	373.89±3.14 ^A^^,^^a^^b^	3.59±0.30 ^A^^,^^a^^b^	0.50±0.09 ^B^^,^^c^
**BE**	68.64±3.85 ^A^^,^^a^^b^	62.30±7.01 ^A^^,^^a^	201.47±1.44 ^B^^,^^b^	360.68±4.60 ^A^^,^^a^^b^	4.03±0.04 ^A^^,^^a^	4.34±0.09 ^A^^,^^a^
**BC**	77.79±6.22 ^A^^,^^a^	67.46±2.51 ^B^^,^^a^	245.95±4.20 ^B^^,^^a^	413.13±2.49 ^A^^,^^a^	3.08±0.01 ^A^^,^^b^	3.55±0.65 ^A^^,^^b^

All results are means ± SD (n = 3).

a-c Same letters indicate no difference between treatments in the same day results within the analysis; different letters indicate difference.

A-B Same letters prescribe that there was no difference between the days results within the analysis; different letters determine the difference.

BN–broiler negative control; BP—broiler positive control (with BHT); BE–broiler meat with *E*. *edulis*; BC–broiler meat with *C*. *brasiliense*

On the first day, BN showed the lowest concentration of TPC, which differed (p<0.05) from the values for BP, BE and BC. This difference is related to the fact that no extract or synthetic antioxidant was added to the control samples. On the other hand, at the end of the storage period (10^th^ day), only the samples treated with natural antioxidants (BE and BC) differed (p<0.05) from the control (BN). The phenolics present in natural extracts are more complex and stable to degradation, whereas it is possible that those that are possibly present in the synthetic antioxidant (BHT) are more labile due to the lower complexity of the additive [40]. Comparing the TPC contents observed at days 0 and 10, BE was the only treatment that did not present a change during this period, whereas the values for BN, BP and BC decreased. This behavior can probably be explained by consumption of phenolic compounds in antioxidant reactions [38], reducing the concentrations of these compounds.

BC also presented the highest concentration of TFC in the first day, which differed (p<0.05) not only from that of the BN but also from those of the BP and BE (p<0.05). The pequi peel probably contains more flavonoids content than that of the juçara waste or the synthetic antioxidants, which explains the higher initial concentration of this compound in this sample. An increase of flavonoid content compared to day 0 was observed in all treatment groups (BN, BP, BE and BC). This increase may be related to the presence of natural compounds in broiler meat and skin [41]. Therefore, during storage, the muscle can undergo changes, such as hydrolysis that result in the development of its own bioactive compounds [42], which can be detected as flavonoids.

The BE treated sample presented the highest concentration of anthocyanins (TAC) on days 0 and 10 compared to the other treatments (BN, BP and BC). The juçara fruit is considered to be a source of anthocyanins, which are responsible for their purple color [14]. However, the BE and BC treated samples showed no differences (p>0.05) as a function of the days of storage whereas in the BN and BP samples, there was a reduction in these compounds. This behavior can be explained by the fact that anthocyanins are consumed during the antioxidant reaction, which reduces the concentrations of these compounds [40].

With respect to the relevant equivalent quantification of antioxidant compounds (TPC, TFC and TAC) in the negative control treatment (BN), this may be related to the fact that the dark musculature of chicken contains compounds with antioxidant properties [40] that can also be detected in these analyses.

### Lipid oxidation stability

The degree of lipid oxidation in the treated samples (BN, BP, BE and BC) was examined using the thiobarbituric acid reagents, and the results are expressed in milligrams of malonaldehyde per gram of meat. All samples showed increases in lipid oxidation during storage (Fig 1B). The values for BN were 2.28 to 4.22, BP 1.64 to 4.01, BE 1.70 to 4.07, and BC 1.78 to 2.89 mgMDA.g^-1^, on day 0 and 10, respectively (Fig 1B). During meat storage, oxidation occurs on double-bond sites in the triacylglycerol molecules in the meat, which results in deterioration that is observed as rancidity [43].

On the first day of storage, the samples treated with the antioxidant compounds had lower lipid oxidation levels than the negative control (BN). On day 2 of storage, BHT was more efficient in reducing oxidation; however, from day 4 until the end of the storage period, the pequi extract presented greater antioxidant action as indicated by the lowest values, 2.52, 2.56, 2.79, 2.89 mgMDA.g^-1^ on days 4, 6, 8, and 10, respectively. The stability of the lipid oxidative process in this treatment was also observed in this period. Pequi extract is a source of antioxidant compounds that act as preservatives against lipid oxidation [5] in refrigerated chicken meat and likely reduced this loss due to oxidation. Therefore, the pequi peel extract proved to be a potential natural antioxidant for use in chicken meat, and possibly in other meat products.

Lipid oxidation is considered to be a process that modifies the chemical properties of molecules, which can lead to loss of function or generation of compounds such as aldehydes and peroxides [43]. Repeated consumption of oxidized fat can pose a health risk to the consumer. However, oxidative stability in complex food such as meat depends on the composition and concentration of reaction substrates, prooxidants and antioxidants compounds content [44]. Therefore, the use of pequi extract to suppress formation of peroxides may be an alternative that could benefits the health of the consumer.

### Protein oxidation stability

On the first day of storage, BE (2.32) and BC (2.12) showed the highest rates of protein oxidation (Fig 1C). However, on day 2, BN (2.64) and BP (2.38) showed greater increases, whereas BC (2.28, 2.33, 2.31, respectively) and BE (2.304, 2.34, 2.40, respectively) remained stable until day 6. At this point, there was a decrease in the carbonyl components was observed. On the last day of storage there were lower protein oxidation rates than those observed at the beginning were observed for BE (1.66) and BC (1.39), whereas BN (1.71) and BP (2.03) presented their highest levels. The degree of protein oxidation in the treatments was determined and reported as the nmol of carbonyl per mg of protein. Generation of carbonyls is the most common form of damage in oxidized proteins [45]. The structural arrangement and amino-acid composition determine the function of proteins, including emulsification, solubility, and gelation, in food matrixes and products. Carbonyl compounds are generated principally by oxidative deterioration of amino acids (lysine, proline, histidine and arginine). These generated compounds interfere with and impair the functions of the meat protein. The addition of antioxidants is one way to reduce this oxidative process in the proteins in food [43].

The behavior of carbonyl compounds during storage can be explained by the fact that carbonyl derivatives can react with lysine amino groups or with proteins or even between different proteins, enabling protein cross-linkages, which can form intra- and intermolecular cross-links [45,46]. These processes reduce the carbonyl concentrations [45,46]. On day 10, BC presented the lowest protein oxidation rates, whereas the pequi extract was shown to have the best ability to stabilize this process. While BP presented the highest rates, showing that this synthetic antioxidant was not shown to have satisfactory applicability for the stabilization of protein oxidation. BE presented similar levels to the control, affirming that the extract of the pequi peel extract had better stabilizing power. Therefore, the use of pequi extract can optimize the conservation of meat products by reducing protein oxidation.

Studies that have examined the effects of the application of natural antioxidants in meat or other products have used rosemary extract, acorn extract, mugwort extract, tropical citrus peel extracts, and rose extract on broiler meat and observed an effect on the stabilization of lipid and protein oxidation. [8–10,47,48] However, the use of juçara wastes and pequi peel extract as a preservative that stabilizes oxidation processes when applied in poultry or other meat are very scarce. Until now, only Frasao [49], on her Ph.D. Thesis, studied these wastes extracts applied in poultry meat. Therefore, this study is innovative, although more research related to this application must be performed.

### Color evaluation

**Fig 3:**
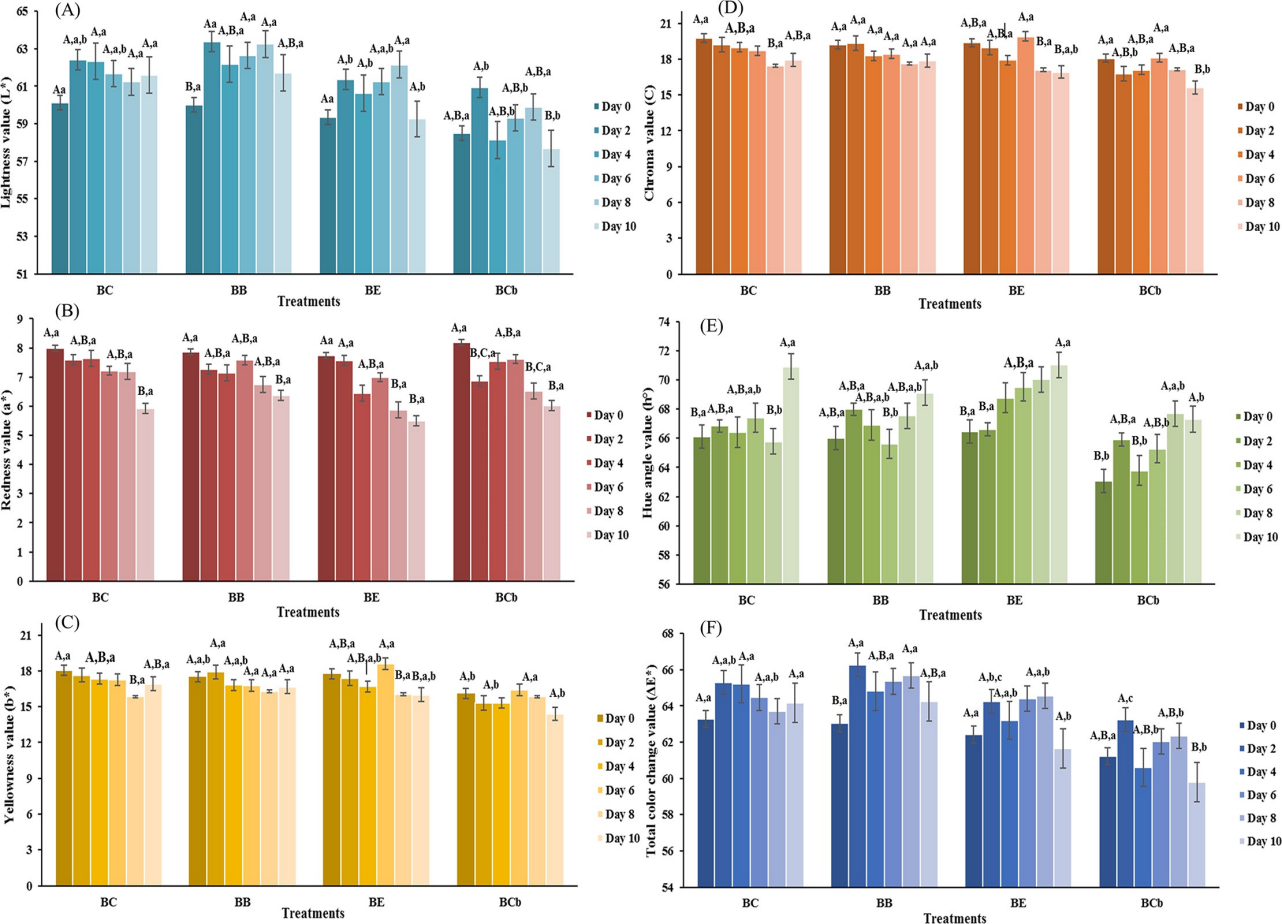
Color parameters of broiler treatments during 10 days of storage at 4°C. All results are means ± SD (n = 3). a-b: the same letters indicate no difference between the treatments in the same day results within the analysis; different letters indicate a difference. A-C: the same letters indicate no difference between the days’ results within the analysis; different letters indicate a difference. BN–broiler negative control; BP—broiler positive control (with BHT); BE–broiler meat with *E*. *edulis*; and BC–broiler meat with *C*. *brasiliense*.

Food color is influenced by various factors including chemical, physical, biochemical, and microbial factors, which can change with microorganism growth, maturation process, post-mortem processing, and storage [47]. In meat, the color is an important attribute that is considered by consumers to characterize the quality of the product, which thus affects the consumer choice. Color characterization of antibiotic-free broiler meat treatments during storage are shown in Fig 3. The lightness (L*), yellowness (b*), and total color change (ΔE*) did not differ (p>0.05) from the beginning to the end of storage time.

Lightness (L*) values did not differ from the beginning to the end of storage time for all treatments. In comparing the treatments, the values did not differ (p>0.05) between BN and BP, however, BE and BC presented lower (p<0.05) values for this parameter than the other two without differences (p>0,05) between them after two days of storage. This behavior can be explained by the fact that extract wastes were added to the BE and BC samples, and the addition of the extracts in these treatments and reaction of compounds led to the browning of the broiler meat. In addition, further deposition of the pigments of these extracts can occur during storage. In particular, the juçara extract contains a high level of anthocyanins [15], and the pequi extract contains carotenoids [36,50]. Anthocyanins are purple [51] pigments and carotenoids are orange [50], and these pigments contributed to the detected darkening.

Redness (a*) values presented no differences (p>0.05) among the treatments, but decreases (p<0.05) were observed during storage for all cases. This reduction in red intensity during the storage of samples can be attributed to myoglobin oxidation, denaturation of hemoglobin and iron oxidation to the ferric form [8,52]. Thus, brownish pigments were formed by chemical degradation, mainly denatured globin hemochromes [8].

The yellowness (b*) values did not differ (p>0.05) from the beginning to the end of storage time for any treatment. However, BC samples presented lower (p<0.05) values for this parameter. This can be explained by the presence of carotenoids pigments, which contribute an orange color [53]. For this measurement, yellow has the most positive value, and orange has lower values [35], which supports this the hypothesis.

Based on the fact that the value of chroma is calculated using the values of a* and b* [35], and thus, the observed decrease (p<0.05) in this value during storage only in the BC treatment is related to the fact that the other treatments presented higher values of b*. This difference, as mentioned previously, may be linked to the presence of carotenoid pigments [53]. In comparing the treatments, at the end of storage BE and BC did not differ (p>0.05), but these two differed (p<0.05) from BN and BP, which were similar (p>0.05). Consistent with the hypothesis presented above, the BE treatment included purple anthocyanin pigments.

Hue angle (h°) is measured in degrees and begins with red (0°), then gradually increases to yellow (90°), green (180°) and blue (270°) [35]. The increase (p<0.05) in the values of this angle for all treatments agrees with the reduction observed in the redness parameter, as indicated by the fact that the values approached degrees between yellow and red (67–72°), consistent with the presence of brownish pigments. With respect to the value of h°, the BC treatment presented the lowest (p<0.05) values. This can be explained by interference by the extract, probably by the carotenes present in the pequi extracts, which can contribute a variation of orange color [35,53].

Regarding the ΔE* is calculated using the parameters L*, a* and b* [35], the BC treatment presented smaller values in two (L* and b*) of these parameters. The total heat change attributed to this treatment was also lower (p<0,05) than those observed in the others. BC presented lower (p<0.05) values of ΔE* after day 2. At the end of storage (day 10) BE presented similar (p>0.05) values of BC, which could be explained by the low values of lightness (L*) that were observed at this time for the treatment with juçara extract.

The color changes in meat can be attributed to muscle structure and pigment concentrations [8,47]. In this study, it is probable that the variations observed with treatment with pequi (BC) and juçara (BE) waste extract can be attributed to the presence of pigments such as carotenoids or anthocyanin, respectively.

### Principal component analysis

**Fig 4:**
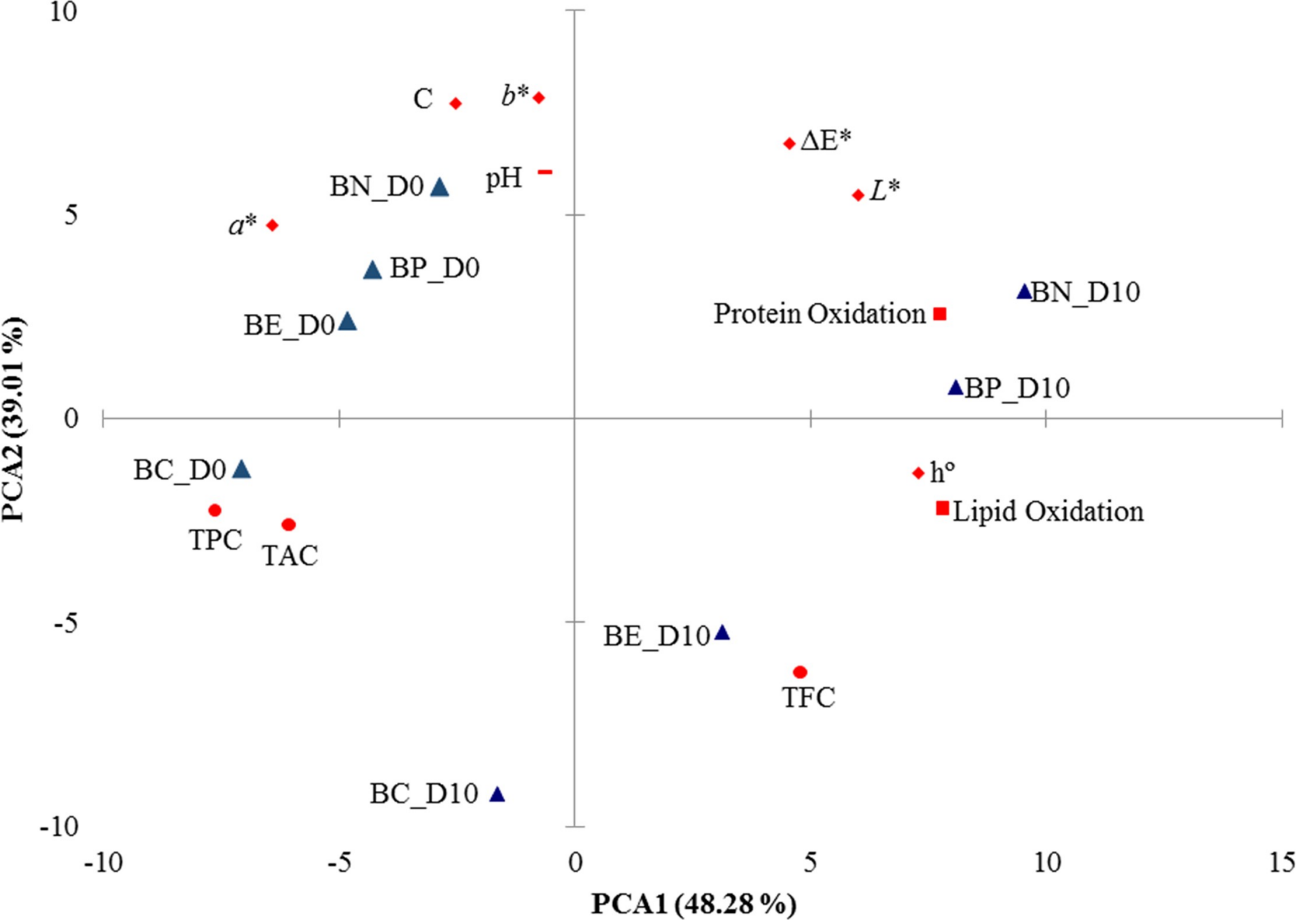
Oxidation processes, antioxidant contents, color parameters, and pH data for antibiotic-free broiler meat treatments on days 0 and 10 of storage in the plane defined by two principal components. Figure was scaled to show subtleties of separation. PCA = principal component analysis, BN–broiler negative control; BP—broiler positive control (with BHT); BE–broiler meat with *E*. *edulis*; BC–broiler meat with *C*. *brasiliense*; D0 = first day of storage; D10 = last day of storage; TPC = total phenolics content; TFC = total flavonoids content; TAC = total anthocyanins content; L* = lightness; a* = redness; b* = yellowness; C = chroma value; h° = hue angle; ΔE* = total color change.

The PCA explained 87.29% of the observed variance, as shown in Fig 4. Principal component-1 (PCA1) was predominant and contributed explained 48.28% of the variance, whereas principal component-2 (PCA2) contributed to 39.01%. PCA1 clustered the treatments by storage period into two groups, day 0 and day 10 of storage, except of BC on day 10. PCA2 clustered the treatments into two groups with or without natural extract addition, i.e., the controls (BN and BP) versus the natural extracts (BE and BC), except BE in the day 0. Moreover, PCA1 with PCA2 clustered the treatments into four different groups: BN, BP and BE on day 0; BN and BP on day 10; BE on day 10; and, BC independent of the day of storage.

In addition, TPC and TAC influenced the BC treatments, whereas on day 10 of storage, BE was influenced by TFC. The treatment with BHT (BP) was influenced by protein oxidation on day 10 of storage and by redness (a*) and pH on day 0. The negative control (BN) was influenced by pH, chroma (C) and yellowness (b*) on day zero of storage and by protein oxidation, lightness (L*) and total color change (ΔE*) on day 10 of storage.

Pearson’s analysis showed strong positive correlations (p < 0.05) between protein and lipid oxidation (r = 0.804), lipid oxidation and angle hue (h°) (r = 0.900), protein oxidation and lightness (L*) (r = 0.876), protein oxidation and angle hue (h°) (r = 0.795), total color change (ΔE*) and protein oxidation (r = 0.756), total color change (ΔE*) and yellowness (b*) (r = 0.740), chroma (C) and redness (a*) (r = 0.758), chroma (C) and yellowness (b*) (r = 0.968), and TPC and TAC (r = 0.761). However, the major negative correlations (p < 0.05) was observed between lipid oxidation and redness (a*) (r = -0.897), lipid oxidation and TPC (r = -0.806), protein oxidation and TPC (r = -0.918), protein oxidation and TAC (r = -0.787), lightness (L*) and TPC (r = -0.822), lightness (L*) and TAC (r = -0.783), redness (a*) and hue angle (h°) (r = -0.888), redness (a*) and TFC (r = -0.844), yellowness (b*) and TFC (r = -0.802), chroma (C) and TFC (r = -0.893), and hue angle (h°) and TPC (r = -0.776).

The positive correlations of protein oxidation with L*, h° and ΔE* were explained by the fact that oxidation of proteins including myoglobin results in discoloration [52]. In addition, the negative correlations between lipid and protein oxidation with TPC and protein oxidation with TAC were related to the antioxidant activities of these compounds [15]. However, the positive correlations observed between ΔE* and b* between chroma and a* and b* were explained by the fact that the calculation of the former parameters utilizes b* and a* [35].

## Conclusions

In conclusion, the direct addition of pequi peel extract was highly effective in the stabilizing the lipid and protein oxidative degradation in pre-oxidized antibiotic-free broiler meat and did not interfere with the total color changes. Therefore, the use of pequi peel as a source of natural antioxidant for application in chicken meat has been proven to be more effective than a synthetic antioxidant (BHT). On the other hand, although less efficient than pequi peel extract, the juçara waste extract can also be used as a technological strategy to reduce the oxidation in antibiotic-free broiler meat for the poultry industry.
